# Supplementary figures and images for: Identification of Key Genes Regulating Sorghum Mesocotyl Elongation through Transcriptome Analysis
Source: Genes (Basel). 2023 Jun 2;14(6):1215. doi: 10.3390/genes14061215 (PMC10298726; doi:10.3390/genes14061215)

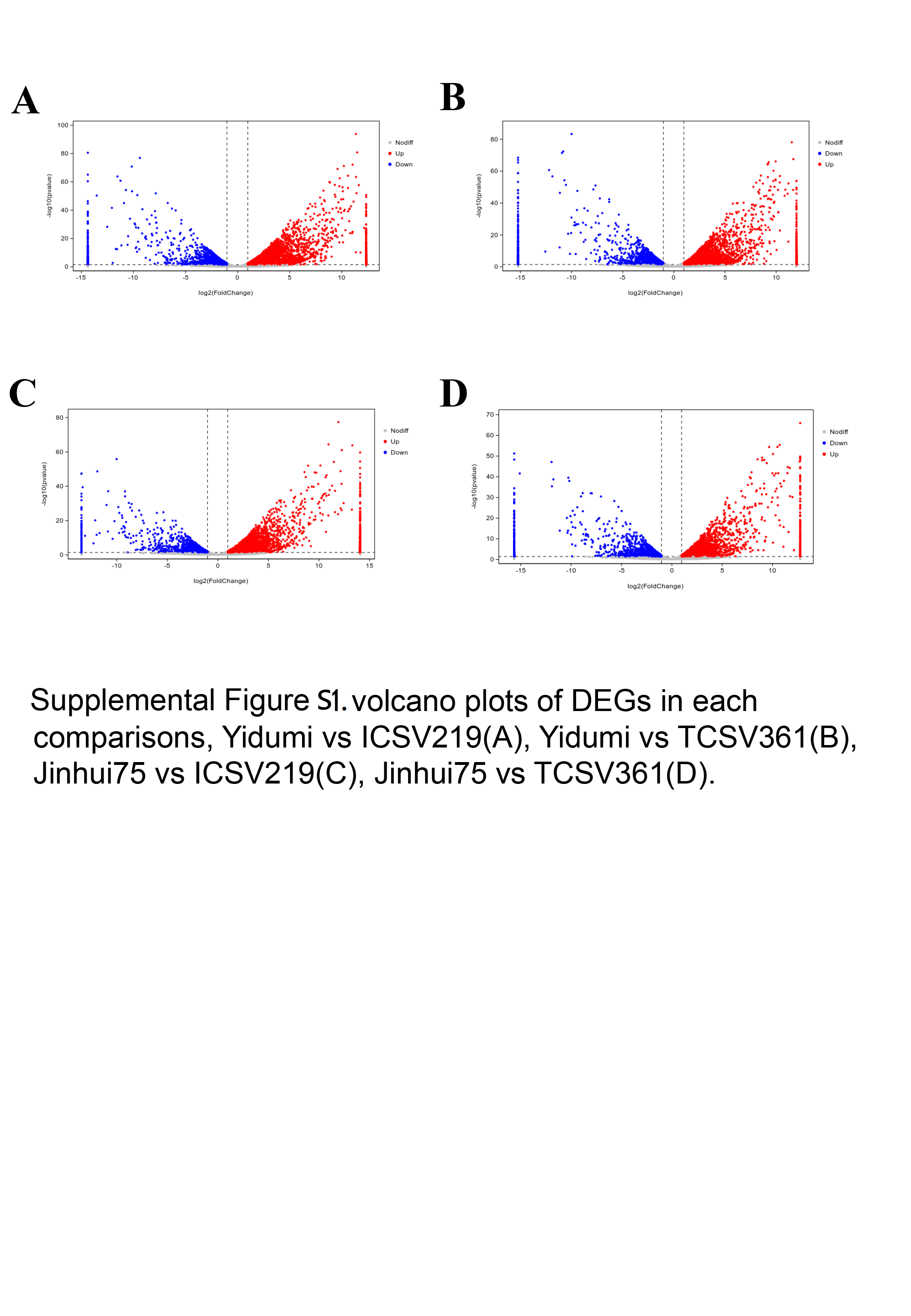

Supplement: Supplementary file 1 [file genes-14-01215-s001.zip › Figure S1.tif]

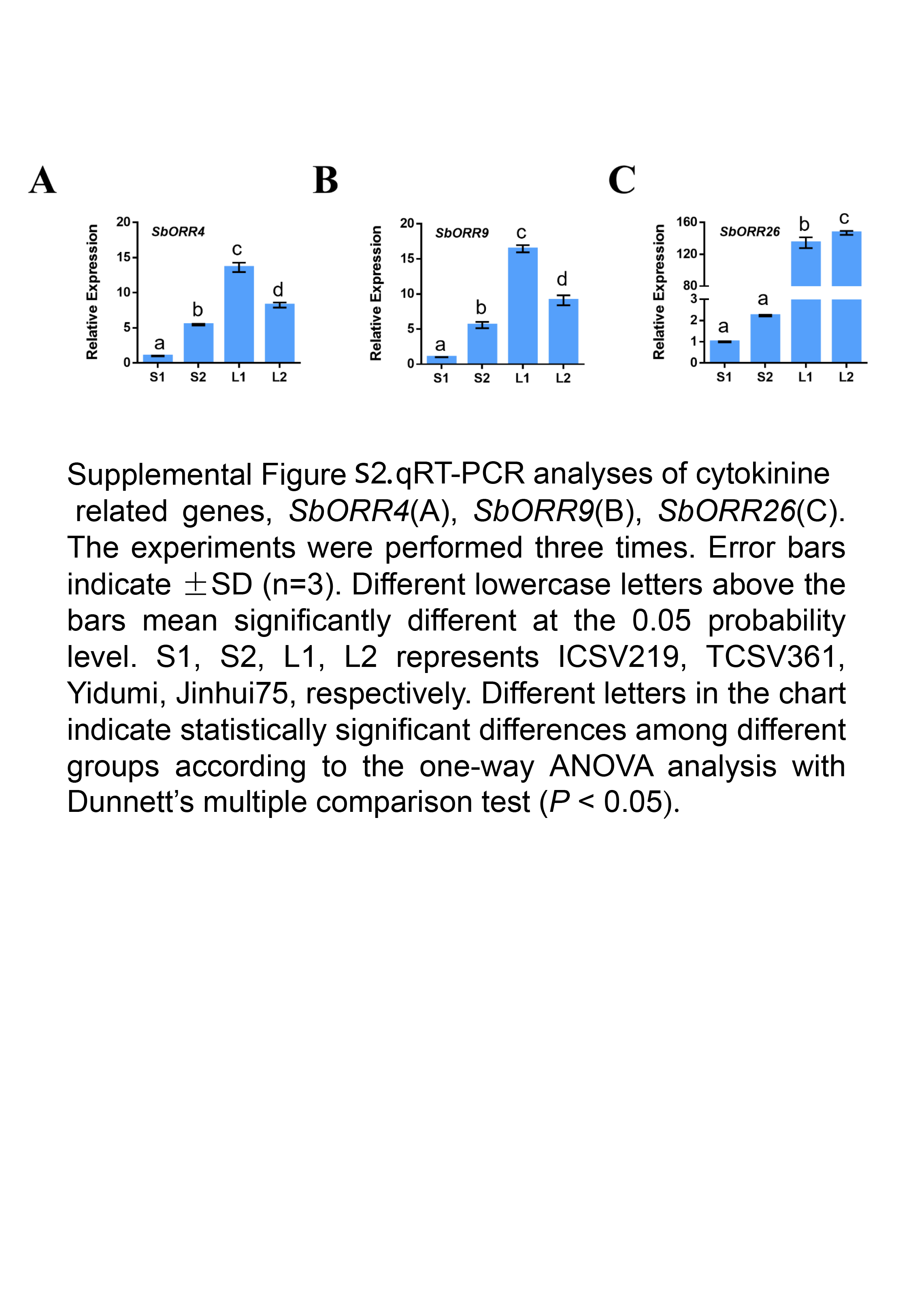

Supplement: Supplementary file 1 [file genes-14-01215-s001.zip › Figure S2.tif]

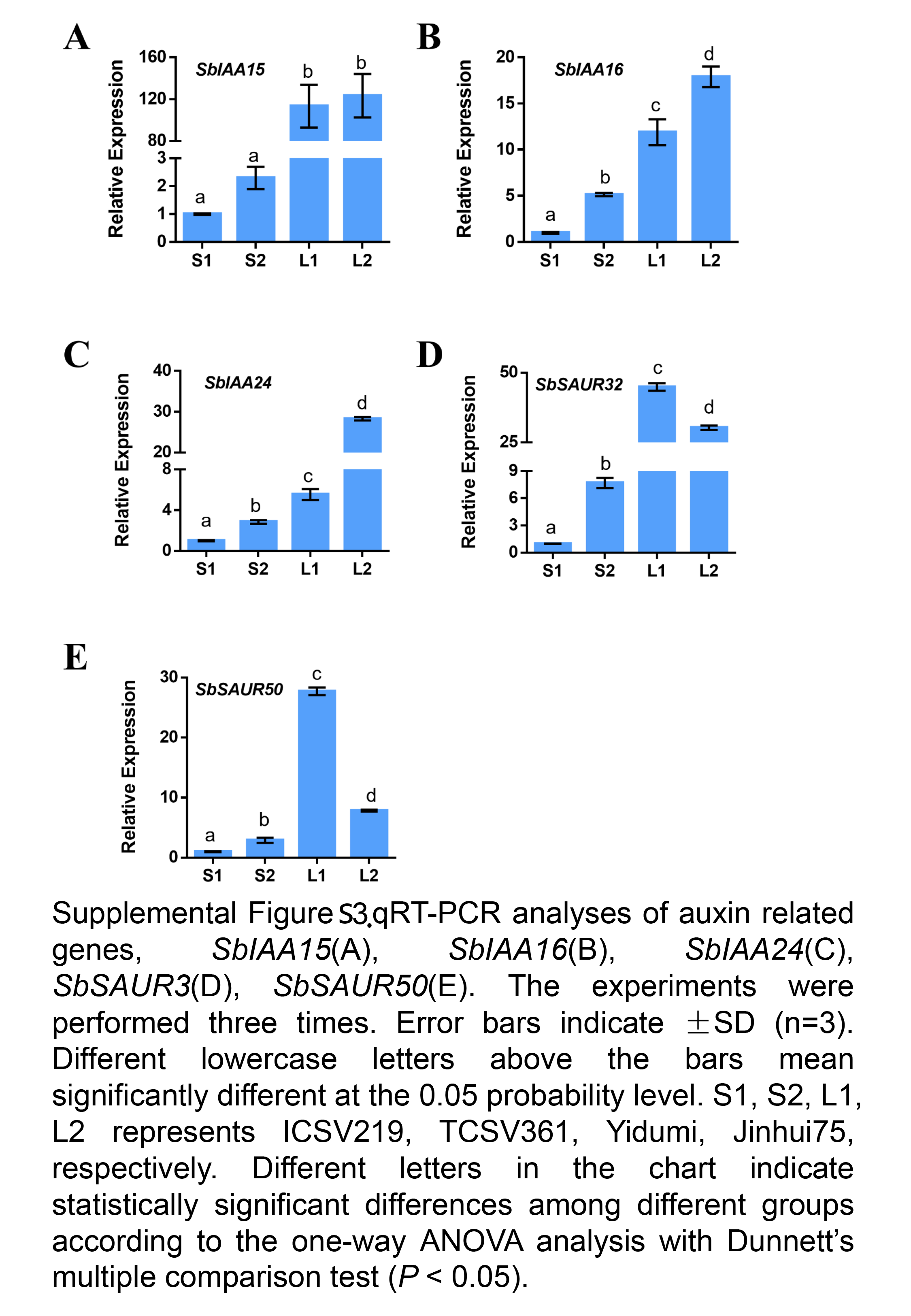

Supplement: Supplementary file 1 [file genes-14-01215-s001.zip › Figure S3.tif]

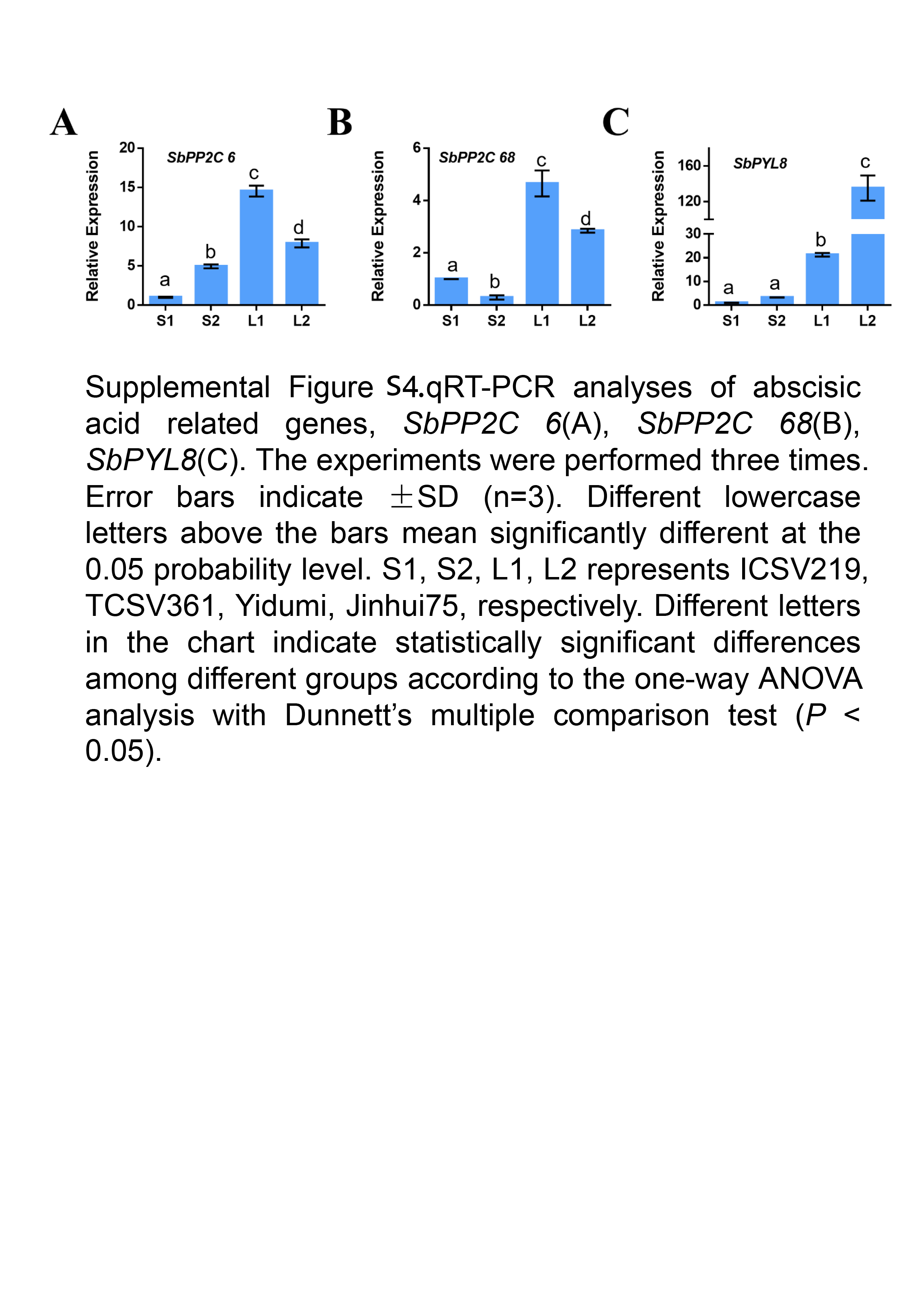

Supplement: Supplementary file 1 [file genes-14-01215-s001.zip › Figure S4.tif]

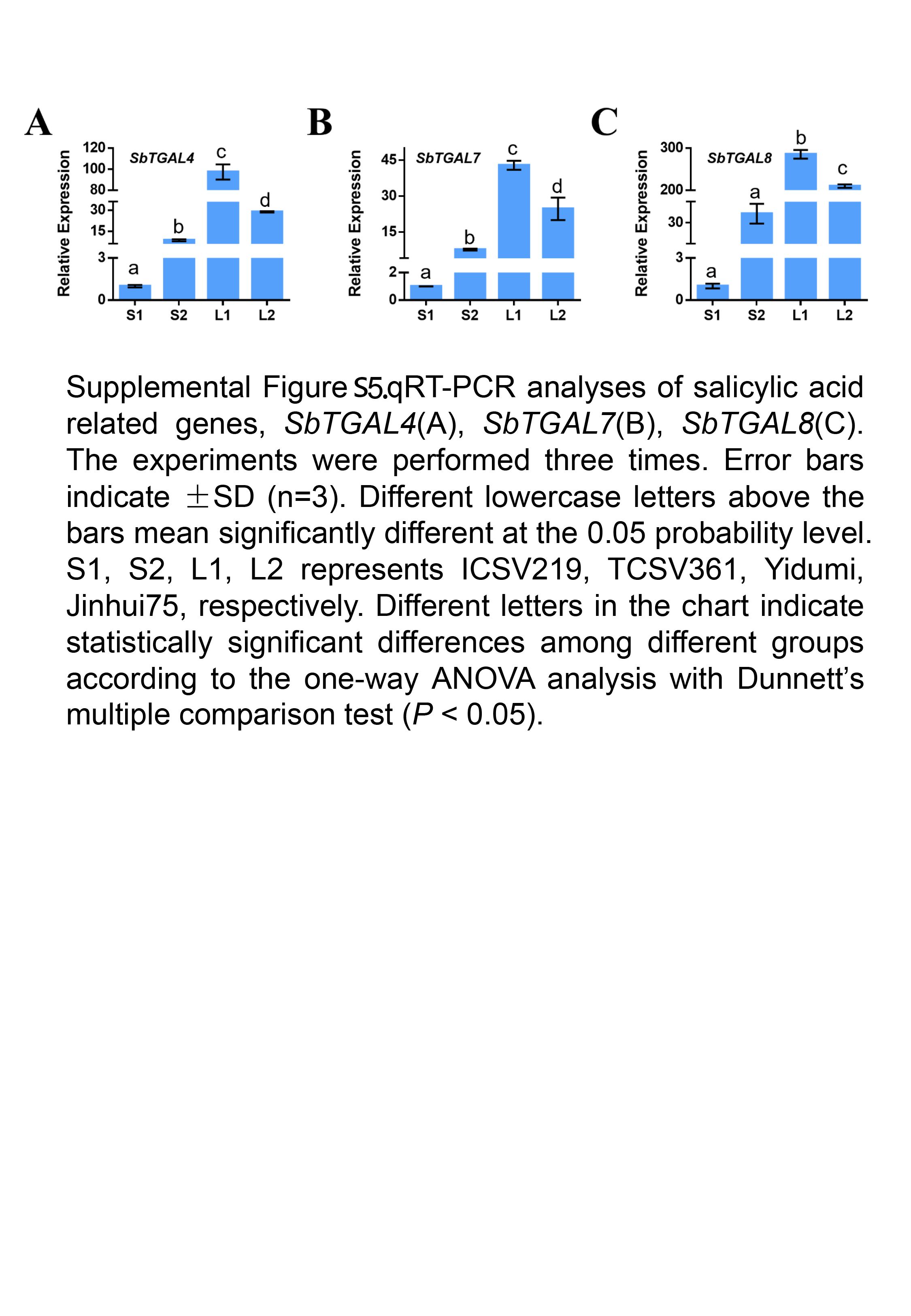

Supplement: Supplementary file 1 [file genes-14-01215-s001.zip › Figure S5.tif]
